# Outbreak and genotyping of canine distemper virus in captive Siberian tigers and red pandas

**DOI:** 10.1038/s41598-017-08462-4

**Published:** 2017-08-15

**Authors:** He Zhang, Fen Shan, Xia Zhou, Bing Li, Jun-Qiong Zhai, Shu-Zhan Zou, Meng-Fan Wu, Wu Chen, Shao-Lun Zhai, Man-Lin Luo

**Affiliations:** 1 Guangdong Provincial Key Laboratory of Prevention and Control for Severe Clinical Animal Diseases, College of Veterinary Medicine, South China Agricultural University, Guangzhou, 510642 China; 2 Guangzhou Zoo, Guangzhou, 510070 China; 3 Animal Disease Diagnostic Center, Institute of Animal Health, Guangdong Academy of Agricultural Sciences, Guangzhou, 510640 China

## Abstract

In this study, four canine distemper virus (CDV) strains were isolated from captive Siberian tigers (*Panthera tigris altaica*) and red pandas (*Ailurus fulgens*) during two separate CDV outbreaks in a zoo in Guangdong province, China. Sequence alignment and phylogenetic analyses based on the full-length hemagglutinin (H) and fusion (F) genes showed that they were closely identical to genotype Asia-1. Prior to confirmation of CDV in Siberian tigers, to control spread of the disease, a live attenuated combination CDV vaccine was used among almost all carnivore animals except for red pandas in which another recombinant combination CDV vaccine was used. However, about two months later, CDV re-emerged and caused the death among red pandas. Based on the vaccination records, the live combination vaccine could be considered an ideal weapon against CDV in zoo carnivore animals. Although the recombinant combination CDV vaccine was safe for red pandas, its protection effectiveness remains to be further investigated. Moreover, according to the outbreak interval time and sequence characterization, we suspected that stray cats circulating in the zoo were the intermediate host, which contributed to CDV spread from stray dogs to zoo animals. This study revealed the importance of vaccination and biosecurity for zoo animals.

## Introduction

Canine distemper virus (CDV) is a small enveloped virus containing a single strand negative-sense RNA molecule about 15.7 kb in size. CDV belongs to the family *Paramyxoviridae* in the order of *Morbillivirus* ^1^. CDV has a wide host range including the families *Urdidae*, *Canidae*, *Procyanidae*, *Hyanidae*, *Felidae*, and *Mustelidae* ^2^. With the change of ecological environment and CDV adaptation of epizootic factors, coupled with the emergence of CDV variants, CDV has novel uncommon hosts, which include non-human primates (such as rhesus monkeys, cynomolgus monkeys, and Japanese macaques)^3–6^, non-carnivore species (such as collared peccaries, wild boars and Sika deer)^7–9^, and marine mammals (such as Lake Baikal seals and Caspian seals)^10–12^. Canine distemper (CD), a “devastating infectious disease” for animals infected with CDV, has a variety of clinical symptoms including sneezing, coughing, runny nose, fever, lethargy, vomiting, diarrhea, and/or loss of appetite. Moreover, the affected animals are easily infected by other pathogenic microorganisms so that CD’s mortality rate is as high as 80%. Recently, there are a number of reports about CDV in wild tigers and zoo tigers^13–19^. CDV infection also caused illness and death in giant pandas^20–22^.

In the present study, we identified two discontinuous CDV outbreaks in captive Siberian tigers (*Panthera tigris altaica*) and red pandas (*Ailurus fulgens*) in a zoo in Guangdong province, China. Moreover, the full-length H and F gene sequences and their corresponding amino acid sequences were characterized and analyzed. Based on the outbreaks of CDV, possible viral origin and future control measures of CDV were discussed in the zoo.

## Results

### CDV confirmation and control

According to clinical manifestations, CDV was considered suspected pathogen during the disease outbreak. Lung and kidney samples of Siberian tiger and red panda were grinded and filtered. Vero cells were inoculated using the supernatants. In generation 3, the obvious cytopathic effect (CPE) was observed. Four CDV strains were thus isolated and named as Siberian tiger/GZZ09/China, Red panda/GZZ1101/China, Red panda/GZZ1102/China and Red panda/GZZ1103/China, respectively. Moreover, they were further confirmed by PCR method. Prior to confirmation of CDV in Siberian tigers, in order to control the spread of the disease among zoo animals, one live attenuated combination CDV vaccine was used among almost all carnivore animals except for red pandas in which a different recombinant combination CDV vaccine was used. Nevertheless, CDV re-emerged only in red pandas about two months later.

### Sequence characterization of H and F genes of CDV

To characterize the four CDV strains, their full length sequences of H gene were obtained. The H gene was composed of 1824 nucleotides, encoding 607 amino acids. Interestingly, they had the same predicted H protein sequences. The H gene sequence of the four stains demonstrated a high nucleotide similarity (99%) with one giant panda-origin strain (GenBank accession number, AF178038) and one tiger-origin strain (KM386683), respectively. However, they were significantly different from the vaccine strains. At the amino acid level, they were 96.5 ~ 96.9% identical to prototype virulent strain A75/17^23^, 92.2% ~ 92.6% to the classical vaccine strain, and 91.9% ~ 92.9% to other vaccine strains. While they had much higher similarity (96.1% ~ 99.8%) with other Asia-1 genotype strains compared with vaccine strains, they differed at positions of 530 and 549. The four current strains had the same amino acid binding sites of SLAM receptor as in several wild isolates including A75/17, one Lynx-origin strain (GU001863) and one canine-origin strain (AF172411) in the same region (Table S1). In addition, they had nine potential glycosylation sites, which were different from other genotypes of CDV strains (Table S2).

Furthermore, the four present CDV strains had the same F gene sequences (i.e., 1989 nucleotides) in length and had high nucleotide identity (99%) with the strains of GZ0803, GZ0804, HeB(07)1, Hebei isolated from dogs, foxes, raccoon dogs and minks, respectively. Meanwhile, at the amino acid level, they had slightly higher identity to wild strains (96.1% ~ 98.5%) than A75/17 (93.9%), Onderstepoort vaccine strain (90.4% ~ 90.5%), and other vaccine strains (90.1% ~ 90.6%). The first 135 amino acids (i.e., the signal peptide sequences) of the F protein were most variable in the CDV genome^24^ and were associated with geographical distribution^25^. In this study, our results revealed that the pre-peptide regions of the present strains were almost identical to each other, but shared only 93.3–97.5% nucleotide and 85.9–96.3% amino acid identity with other Asia-1 strains, respectively. In particular, the identity with Onderstepoort vaccine strain was as low as 81% and 63.7% at the nucleotide level and at the amino acid level, respectively.

### Phylogenetic analysis of H and F genes of CDV

**Figure 1:**
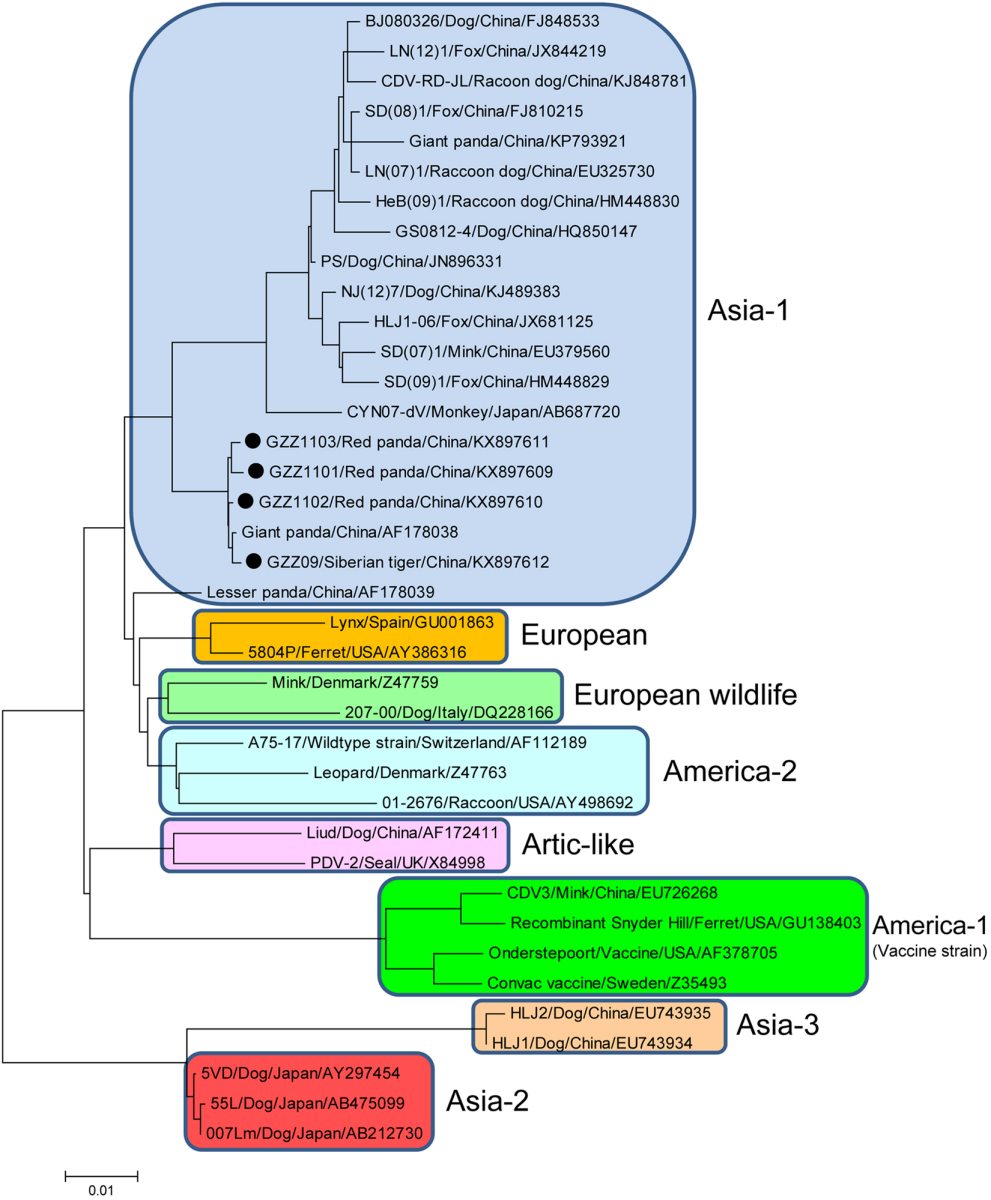
Phylogenetic analysis based on H gene sequences of different genotypes of CDVs. Note: The CDV strains in this study were marked with “•”.

**Figure 2:**
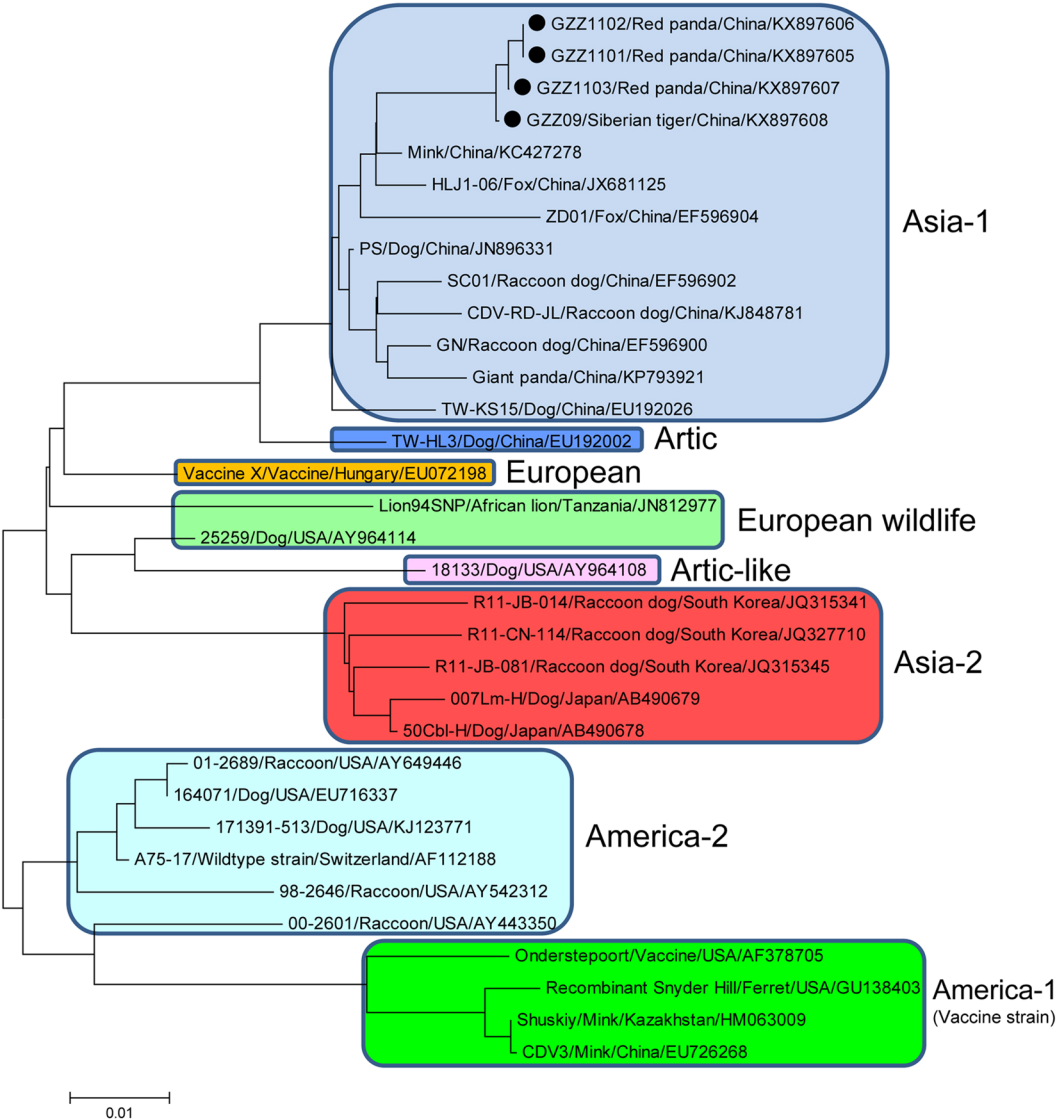
Phylogenetic analysis based on F gene sequences of different genotypes of CDVs. Note: The CDV strains in this study were marked with “•”.

To determine genetic relationships of those CDVs, phylogenetic analyses based on the H and F gene sequences were performed. The results revealed that Siberian tiger/GZZ09/China, Red panda/GZZ1101/China, Red panda/GZZ1102/China and Red panda/GZZ1103/China were categorized as Asia-1 genotype corresponding to the present pandemic genotype, but were significantly distinct from Asia-2, Asia-3, and other genotypes (Figs 1 and 2). In addition, a small subgenotype branch was formed within the Asia-1 genotype, mainly resulted from the recent identified strains (Figs 1 and 2).

### Advanced structural analysis of H and F proteins

Advanced structural profiles of H and F proteins were predicted. For H protein, there were two main differences between Siberian tiger-origin CDV strain and red panda-origin CDV strain (Fig. S1), which shared similar structural profiles to Onderstepoort, CDV3, Convac and Recombinant Snyder Hill, respectively. However, for F protein, compared with those of CDV vaccine strains, CDV strains of Siberian tiger-origin and red panda-origin had specific structural profiles (Fig. S2).

## Discussion

In the past few years, there were about 500 wild Siberian tigers remaining in the world, which are mainly distributed in the Russian Far East (*n* ≈ 480) and northeast China (*n* ≈ 20)^17^. Due to human population expansion, illegal hunting and environmental changes, Siberian tigers have rarely been observed in the wild. According to the statistics, fewer than 10, 000 red pandas remain in the wild. At present, Siberian tigers and red pandas have been listed as endangered species. They were often captive in the zoos or wildlife rescue stations in order to expand the numbers of the population. Under the circumstance, they are considered special animals (e.g. having high ornamental value) with enough food and without natural enemies; however, some serious plagues including CD often threaten their lives. Since 2010, the number of CDV outbreaks has increased in wild Siberian tigers in Russia, leading to the reduction of wild Siberian tiger population^14, 16–19^. In this study, we reported the death of captive Siberian tigers and red pandas due to the infection of wild-type (Asia-1) CDV strains in China, suggesting that CDV poses a significant threat to zoo animals.

At present, there are variety of available CDV vaccines (Table 1) against CD in China. Most of them are used for dogs, and only two vaccines are used for farmed fur animals (minks and foxes). Vaccination is an effective way to control CD in dogs^26^. However, for endangered animals (such as Siberian tigers, south China tigers and red pandas), zoo owners are quite cautious to use the live attenuated canine-origin vaccines^27, 28^. In 2006, one study showed that one live attenuated combination vaccine (including CDV, strain Onderstepoort) (Intervet International B.V.) was safe and effective in zoo large felids including Siberian tigers, African lion (*Panthera leo*) and Eurasian lynx (*Lynx lynx*); while it could not produce CDV neutralizing antibody after vaccination in white tigers (*Bengal White Tiger*)^29^. In 1976, Onderstepoort vaccine strain-induced canine distemper was reported in red pandas^30^. Thus, the CDV vaccines have not been used for emergency vaccination in the zoo until the recent outbreak. Considering virulence residue of vaccine strains, a recombinant combination CDV vaccine (strain Onderstepoort) was introduced and used in red pandas. Compared with the live combination CDV vaccine used in zoo carnivore animals, the recombinant CDV vaccine is safe for red pandas, but its protection effectiveness remains to be unknown. Actually, the recombinant canarypox-vectored CDV vaccine was found to only induce low levels of neutralizing antibody in fewer captive tigers at 66-day of post vaccination^31^, indicating that the recombinant CDV vaccine was not a good choice for emergency vaccination. To a certain extent, the re-emergence of CDV in red pandas being administered with the recombinant CDV vaccine in the present study confirmed the theory. Although the sequence characterization (Tables S1 and S2) and advanced structural profiles (Figures S1 and S2) of H and F proteins of the present strains were different from that of the live vaccine strains (including Onderstepoort), the vaccine had good emergency prevention effects for zoo carnivore animals except for red pandas, it suggested that the Onderstepoort strain could provide adequate protection for most carnivore animals^29^. However, for endangered animal species including red pandas and white tigers, it is necessary to develop effective and species-specific CDV vaccines, and evaluate corresponding vaccination strategies. Nowadays, despite of available CDV vaccines, due to high cost and short protection period, vaccination was not routinely carried out in many places, particularly in less economically developed countries and regions. In China, there were large number of companion dogs, stray dogs and carnivores, Chinese government and veterinary practitioners still face considerable challenges for the control of CD.

**Table 1:** Lists of CDV vaccines used in China.

Vaccine type	Vaccine component	Manufacturer	Use range
Combination vaccine	Live Vaccine against Canine Distemper, Adenovirus Type 2, Parvovirosis, Parainfluenza Virus, and Inactivated Vaccine Against Canine Leptospirosis (Canicola + Icterohaemorrhagiae)	Laboratorios HIPRA S.A., Spain	Dog
Combination vaccine	Live Vaccine against Canine Distemper, Adenoviroses, Parvovirosis and Parainfluenza Type 2, and Inactivated Vaccine Against Leptospira Canicola And Leptospira Icterohaemorrhagiae Leptospiroses	Merial SAS, France	Dog
Combination vaccine	Live Vaccine against Canine Distemper, Adenovirus Type 2, Parainfluenza, and Killed Vaccine against Leptospira Canicola and Icterohaemorrhagiae Bacterin	Zoetis Inc., Lincoln, USA	Dog
Combination vaccine	Live Vaccine against Canine Distempe, Adenovirus Type 2, Parainfluenza and Parvovirus	Zoetis Inc., Lincoln, USA	Dog
Combination vaccine	Live Vaccine against Canine Distempe and Parvovirus	Intervet International B.V., the Netherlands	Dog
Combination vaccine	Live Vaccine against Canine Distemper, Adenovirus, Parvovirus, Parainfluenza	Intervet International B.V., the Netherlands	Dog
Combination vaccine	Live Vaccine against Canine Distemper, Rabies, Adenovirus Type 2, Parainfluenza and Parvovirus	Yangling Lvfang Bio. Co., Ltd. China; Jilin Five-star Animal Health pharmaceutical, factory, China	Dog
Univalent vaccine	Live Vaccine against Canine Distemper (Strain CDV-11)	Qilu Animal Health Products, Co., Ltd. Jinan, China	Fox
Univalent vaccine	Live Vaccine against Canine Distemper (Strain CDV3-CL)	Jilin Teyan Bio-Tec. Co., Ltd; Jilin Zhongte Bio-Tec. Co., Ltd. China	Mink

Previous studies demonstrated that possible sources of CDV in zoo outbreaks were small carnivores (such as raccoons, wild raccoon dogs), which may come in contact with captive cats. CDV spread through aerosol droplets and contact with the secretions of infected animals^15, 32^. The zoo mentioned in this study, located in the downtown area (where there are about 100, 000 stray dogs and 60, 000 stray cats), receives many visitors every day. These stray animals that are lacking of the vaccination could pose a serious threat to the health of domesticated pets, zoo animals and even humans. CDV could infect domesticated cats (*Felis domesticus*) without resulting in significant clinical diseases and death in the infected cats^33–37^. In this study, the living area of Siberian tigers is near to the downtown street where stray dogs and cats run free and stray dogs could not enter the zoo. However, stray cats that have good climbing ability could access freely to the zoo. Therefore, according to the interval time between the two outbreaks, we suspected that stray cats circulating in the zoo were the intermediate host and they contributed to CDV spread from stray dogs to zoo animals. Online Blastn results showed that four current CDV F gene sequences had the highest similarity (only one- or two-nucleotide different) with one canine-origin CDV sequence (GZ0804, KC667068) that originated from the same location in 2012. This information further supported that the possible source of CDV in the present outbreaks were stray dogs or pet dogs. Therefore, biosecurity issue should be taken seriously in the zoo. For example, the zoo should construct some closed living areas with the viewing platforms for susceptible animals to avoid contact with stray cats. Moreover, the zoo should strengthen the management of tourists (e. g., not to throw food to animals) and animal keepers (e. g., to emphasize to the need of personal disinfection).

In summary, the present study reported the outbreaks of CDV (Asia-1 genotype) in captive Siberian tigers and red pandas in a zoo in Guangdong province, Southern China, and further revealed the importance of routine vaccine immunization and biosecurity for zoo animals.

## Materials and Methods

### Samples

In September 2015, one subadult Siberian tiger (about 18 months old) in a zoo of Guangdong province was characterized by mental depression, ataxia, diarrhea, accompanied by clinical symptoms (including coughing and thick mucus coming from the eyes and nose) of respiratory diseases, and finally died. Canine distemper virus was suspected as the pathogen of the outbreak. To prevent the spread of the disease, an emergency vaccination was performed using a live attenuated combination CDV vaccine (against CDV, canine adenovirus, canine parvovirus, canine parainfluenza virus) (Intervet International B.V., Netherlands) among all carnivore animals except for red pandas. Red pandas were vaccinated by another combination vaccine containing a lyophilized suspension of a recombinant canarypox vector expressing the HA and F glycoproteins of CDV, modified live canine adenovirus type 2, and canine parvovirus (Merial, Inc., Duluth, GA, USA). Unfortunately, about two months later, five red pandas also successively died because of suspicious CDV infection. The living areas of Siberian tigers are about 200 meters far away from the living areas of red pandas. Lung and kidney samples were collected from the died animals and stored at −80 °C until use.

### Cells

Vero cells were grown (at 37 °C in 5% CO_2_) in Dulbecco’s Modified Eagle’s Medium (DMEM, GIBCO, Grand Island, NY, USA) supplemented with 10% heat-inactivated fetal calf serum (FBS, Gibco® by life technologies, South America), 50 U/ml penicillin, and 50 μg/ml streptomycin (GIBCO). Then, Vero cells were inoculated with tissue filtrates and incubated until cytopathic effect (CPE) was observed.

### Viral RNA extraction and RT-PCR detection

Viral RNA was extracted from cell supernatants using E. Z. N. A Viral RNA Mini kit (OMEGA, USA), and reverse-transcription (RT) was performed with random 9-mer primers using TaKaRa RNA LA PCR kit Ver.1.1 (Takara, Japan) at 30 °C for 10 min, 42 °C for 30 min, 70 °C for 15 min and 5 °C for 5 min. PCR detection was performed using a set of primers targeting partial H gene (Table 2). PCR reaction conditions were used as followed: 94 °C for 5 min, and 30 cycles of amplification (denaturation at 94 °C for 30 sec, annealing at 56 °C for 30 sec, and extension at 72 °C for 1 min), and a final extension at 72 °C for 15 min. The expected product size was 587 bp.

**Table 2:** PCR primers used in this study.

Primers	Target	Expected size
CDV-HF: 5′-ATAGATGTCTTGACACCGCTCTT-3′	Partial H gene	587 bp
CDV-HR: 5′-GTACATACCTTGGCTTTGGAACT-3′		
CDV-HC-F: 5′-TCGAAATCCTATGTGAGATCACT-3′	Complete H gene	2098 bp
CDV-HC-R: 5′-ATGCTGGAGATGGTTTAATTCAATCG-3′		
CDV-FC-F: 5′-CAGACAAGCCCCATGCACAA-3′	Complete F gene	2153 bp
CDV-FC-R: 5′-TGGACTACCTGAGYCCTAAGT-3′		

### Amplification of complete H and F gene of CDV

To further characterize those CDV strains, their complete H and F gene sequences were amplified using two pairs of PCR primers (Table 2), respectively^38, 39^. RT process was same as described above. PCR reaction conditions of H gene were as the followings: 94 °C for 10 min, 35 cycles of amplification (denaturation at 94 °C for 60 sec, annealing at 54 °C for 60 sec, and extension at 72 °C for 3 min), and a final extension at 72 °C for 10 min. The PCR reaction conditions of F gene were: 94 °C for 10 min, 35 cycles of amplification (denaturation at 94 °C for 60 sec, annealing at 52 °C for 60 sec, and extension at 72 °C for 3 min), and a final extension at 72 °C for 10 min. PCR products were cloned into pMD18-T vector (Takara, Japan), positive recombinant plasmids were purified using QIAquick Gel Extraction Kit (QIAGEN GmbH, Hilden, Germany), and sequenced by sanger sequencing method (BGI Shenzhen company).

### Sequence analysis

The gene sequences were spliced by SeqMan program (Lasergene software Version 7.1). Four H and four F gene sequences of CDV were submitted into GenBank database (GenBank accession numbers: KX897605-KX897612). Nucleotide and amino acid sequences were compared by MegAlign. The phylogenetic trees based on H and F genes (Table S3) were constructed using Neighbor-joining method (1,000 replications) implemented in MEGA 5.1 software. Moreover, the N-glycosylation sites of H protein were analyzed with NetNGlyc 1.0 Server (http://www.cbs.dtu.dk/services/NetNGlyc/). The threshold value was set at 0.32.

### Structure prediction of H and F proteins

To master the advanced structure feature of H and F protein, online prediction program (Swiss-Model) was used (https://swissmodel.expasy.org/interactive). Their structure was displayed using the CPK style in a molecule window (Discovery Studio 4.5 software).

## Electronic supplementary material

Supplementary information
