# Supplementary material for: Outbreak and genotyping of canine distemper virus in captive Siberian tigers and red pandas
Source: Sci Rep. 2017 Aug 15;7:8132. doi: 10.1038/s41598-017-08462-4 (PMC5557937; doi:10.1038/s41598-017-08462-4)
Supplement: Supplementary file 1 — Supplementary information [file 41598_2017_8462_MOESM1_ESM.doc]

**Supplemental files**

**Outbreak and genotyping of canine distemper virus in captive Siberian tigers and red pandas**

He Zhang^1^, Fen Shan^2^, Xia Zhou^1^, Bing Li^1^, Jun-Qiong Zhai^1^, Shu-Zhan Zou^1^, Meng-Fan Wu^1^, Wu Chen^2,*^, Shao-Lun Zhai^3,*^, Man-Lin Luo^1,*^

**Author's affiliation:**

^1^ Guangdong Provincial Key Laboratory of Prevention and Control for Severe Clinical Animal Diseases, College of Veterinary Medicine, South China Agricultural University, Guangzhou 510642, China;

^2^ Guangzhou Zoo, Guangzhou 510070, China;

^3^ Animal Disease Diagnostic Center, Institute of Animal Health, Guangdong Academy of Agricultural Sciences, Guangzhou 510640, China

**^*^ Corresponding author**

Guangzhouchenwu@sina.com; zhaishaolun@163.com; 710510116@qq.com

**Supplemental Table S1** Analysis of binding sites of SLAM receptor with different CDV strains

| Genotype | strain | GenBank | 525 | 526 | 529 | 530 | 549 |
| --- | --- | --- | --- | --- | --- | --- | --- |
| Asia-1 | Giant panda/SX/2014/China | KP793921 | Y | D | R | G | H |
|  | Raccoon dog/LN(07)1/China | EU325730 | Y | D | R | G | Y |
|  | Fox/SD(08)1/China | FJ810215 | Y | D | R | G | Y |
|  | Raccoon dog/CDV-RD-JL/China | KJ848781 | Y | D | R | G | Y |
|  | Lupus/BJ080326/China | FJ848533 | Y | D | R | G | Y |
|  | Fox/LN(12)1/China | JX844219 | Y | D | R | G | Y |
|  | Raccoon dog/HeB(09)1/China | HM448830 | T | D | R | G | Y |
|  | Dog/GS0812-4/China | HQ850147 | Y | D | R | G | Y |
|  | Dog/PS/China | JN896331 | Y | D | R | G | Y |
|  | Dog/NJ(12)7/China | KJ489383 | Y | D | R | G | Y |
|  | Fox/HLJ1-06/China | JX681125 | Y | D | R | G | Y |
|  | Mink/SD(07)1/China | EU379560 | Y | D | R | G | H |
|  | Fox/SD(09)1/China | HM448829 | Y | D | R | G | H |
|  | Monkey/CYN07-dV/Japan | AB687720 | Y | D | R | G | Y |
|  | Giant panda/China | AF178038 | Y | D | R | G | Y |
|  | Lesser panda/China | AF178039 | Y | D | R | D | Y |
|  | Siberian tiger/GZZ09/China | KX897612 | Y | D | R | G | Y |
|  | Red panda/GZZ1101/China | KX897609 | Y | D | R | G | Y |
|  | Red panda/GZZ1102/China | KX897610 | Y | D | R | G | Y |
|  | Red panda/GZZ1103/China | KX897611 | Y | D | R | G | Y |
| Asia-2 | Dog/55L/Japan | AB475099 | Y | D | R | E | Y |
|  | Dog/007Lm/Japan | AB212730 | Y | D | R | E | Y |
|  | Dog/5VD/Japan | AY297454 | Y | D | R | E | Y |
| Asia-3 | Dog/HLJ1/China | EU743934 | Y | D | R | R | Y |
|  | Fox/HLJ2/China | EU743935 | Y | D | R | R | Y |
| European | Ferret/5804P/USA | AY386316 | Y | D | R | G | H |
|  | Lynx/Spain | GU001863 | Y | D | R | G | Y |
| European  (wildlife) | Dog/207-00/Italy | DQ228166 | Y | D | R | N | H |
|  | Mink/Denmark | Z47759 | N | D | R | D | Y |
| America-2 | Leopard/Denmark | Z47763 | Y | D | R | G | H |
|  | Raccoon/01-2676/USA | AY498692 | Y | D | R | R | H |
|  | A75-17/Switzerland | AF112189 | Y | D | R | G | Y |
| Artic-like | Dog/liud/China | AF172411 | Y | D | R | G | Y |
|  | Seal/PDV-2/UK | X84998 | Y | D | R | N | Y |
| America-2  (Vaccine  Strain) | Onderstepoort/USA | AF378705 | Y | D | R | S | H |
|  | Convac vaccine/Sweden | Z35493 | Y | D | R | S | H |
|  | Mink/CDV3/China | EU726268 | Y | D | R | N | H |
|  | Ferret/Recombinant Snyder Hill/USA | GU138403 | Y | D | R | N | Y |

**Supplemental Table S2** Distribution status of N-glycosylation sites of H protein in different genotypes of CDVs

| Genotype | CDV strain | GenBank | 19-21 | 149-151 | 309-311 | 391-393 | 422-424 | 456-458 | 584-586 | 587-589 | 603-605 |
| --- | --- | --- | --- | --- | --- | --- | --- | --- | --- | --- | --- |
| Asia-1 | Giant panda/SX/2014/China | KP793921 | Y | Y | Y | Y | Y | Y | Y | Y | Y |
|  | Raccoon dog/LN(07)1/China | EU325730 | Y | Y | Y | Y | Y | Y | Y | Y | Y |
|  | Fox/SD(08)1/China | FJ810215 | Y | Y | Y | Y | Y | Y | Y | Y | Y |
|  | Raccoon dog/CDV-RD-JL/China | KJ848781 | Y | Y | Y | Y | Y | Y | Y | Y | Y |
|  | Fox/LN(12)1/China | JX844219 | Y | Y | Y | Y | Y | Y | Y | Y | Y |
|  | Raccoon dog/HeB(09)1/China | HM448830 | Y | Y | Y | Y | Y | Y | Y | Y | Y |
|  | Dog/GS0812-4/China | HQ850147 | Y | Y | Y | Y | Y | Y | Y | Y | Y |
|  | Dog/NJ(12)7/China | KJ489383 | Y | Y | Y | Y | Y | Y | Y | Y | Y |
|  | Fox/HLJ1-06/China | JX681125 | Y | Y | Y | Y | Y | Y | Y | Y | Y |
|  | Mink/SD(07)1/China | EU379560 | Y | Y | Y | Y | Y | Y | Y | Y | Y |
|  | Fox/SD(09)1/China | HM448829 | Y | Y | Y | Y | Y | Y | Y | Y | Y |
|  | Dog/PS/China | JN896331 | Y | Y | Y | Y | Y | Y | Y | Y | Y |
|  | Monkey/CYN07-dV/Japan | AB687720 | Y | Y | Y | Y | Y | Y | Y | Y | Y |
|  | Giant panda/China | AF178038 | Y | Y | Y | Y | Y | Y | Y | Y | Y |
|  | Lesser panda/China | AF178039 | Y | Y | Y | Y | Y | Y | N | Y | Y |
|  | Siberian tiger/GZZ09/China | KX897612 | Y | Y | Y | Y | Y | Y | Y | Y | Y |
|  | Red panda/GZZ1101/China | KX897609 | Y | Y | Y | Y | Y | Y | Y | Y | Y |
|  | Red panda/GZZ1102/China | KX897610 | Y | Y | Y | Y | Y | Y | Y | Y | Y |
|  | Red panda/GZZ1103/China | KX897611 | Y | Y | Y | Y | Y | Y | Y | Y | Y |
| Asia-2 | Dog/007Lm/Japan | AB212730 | Y | Y | Y | Y | Y | Y | N | Y | Y |
|  | Dog/55L/Japan | AB475099 | Y | Y | Y | Y | Y | Y | N | Y | Y |
|  | Dog/5VD/Japan | AY297454 | Y | Y | Y | Y | Y | Y | N | Y | Y |
| Asia-3 | Dog/HLJ1/China | EU743934 | Y | Y | Y | N | Y | Y | N | Y | Y |
|  | Fox/HLJ2/China | EU743935 | Y | Y | Y | N | Y | Y | N | Y | Y |
| European | Ferret/5804P/USA | AY386316 | Y | Y | Y | Y | Y | Y | N | Y | Y |
|  | Lynx/Spain | GU001863 | Y | Y | Y | Y | Y | Y | N | Y | N |
| European  (wildlife) | Mink/Denmark | Z47759 | Y | Y | Y | Y | Y | Y | N | Y | Y |
|  | Dog/207-00/Italy | DQ228166 | Y | Y | Y | Y | Y | Y | N | N | Y |
| America-2 | Leopard/Denmark | Z47763 | Y | Y | Y | Y | Y | Y | N | Y | N |
|  | A75-17/Switzerland | AF112189 | Y | Y | N | Y | Y | Y | N | Y | Y |
|  | Raccoon/01-2676/USA | AY498692 | N | Y | Y | Y | Y | Y | N | Y | Y |
| Artic-like | Dog/liud/China | AF172411 | Y | Y | Y | Y | Y | Y | N | Y | Y |
|  | Seal/PDV-2/UK | X84998 | Y | Y | Y | Y | Y | Y | N | Y | Y |
| America-2  (Vaccine  Strain) | Onderstepoort/USA | EU143737 | Y | Y | N | N | Y | N | N | Y | N |
|  | Convac vaccine/Sweden | Z35493 | Y | Y | N | Y | Y | Y | N | Y | Y |
|  | Mink/CDV3/China | EU726268 | Y | Y | N | Y | Y | Y | N | Y | Y |
|  | Ferret/Recombinant Snyder Hill/USA | GU138403 | N | Y | N | Y | Y | Y | N | Y | Y |

Note: Y. N-glycosylation sites; N. Negative

**Supplemental Table S3** Information of CDV sequences used in this study

| Gene | Strain name | Source | Country | Year | GenBank Nos. | Nucleotide (nt) |
| --- | --- | --- | --- | --- | --- | --- |
| CDV-H | LN(07)1 | Raccoon dog | China | 2007 | EU325730 | 1824 |
|  | Giant panda/SX/2014 | Giant panda | China | 2014 | KP793921 | 1824 |
|  | SD(08)1 | Fox | China | 2008 | FJ810215 | 1824 |
|  | CDV-RD-JL | Raccoon dog | China | 2014 | KJ848781 | 1824 |
|  | BJ080326 | Dog | China | 2008 | FJ848533 | 1824 |
|  | LN(12)1 | Fox | China | 2012 | JX844219 | 1824 |
|  | HeB(09)1 | Raccoon dog | China | 2009 | HM448830 | 1824 |
|  | GS0812-4 | Domestic dog | China | 2008 | HQ850147 | 1824 |
|  | PS | Dog | China | 2010 | JN896331 | 1824 |
|  | NJ(12)7 | Dog | China | 2012 | KJ489383 | 1824 |
|  | HLJ1-06 | Fox | China | 2006 | JX681125 | 1824 |
|  | SD(07)1 | Mink | China | 2007 | EU379560 | 1824 |
|  | SD(09)1 | Fox | China | 2009 | HM448829 | 1824 |
|  | CYN07-dV | Monkey | Japan | 2008 | AB687720 | 1824 |
|  | * | Giant panda | China | * | AF178038 | 1824 |
|  | * | Lesser panda | China | 1999 | AF178039 | 1824 |
|  | 5804P | Ferret | USA | 2003 | AY386316 | 1824 |
|  | * | Lynx | Spain | 2005 | GU001863 | 1824 |
|  | * | Leopard | Denmark | 1997 | Z47763 | 1824 |
|  | 01-2676 | Raccoon | USA | * | AY498692 | 1824 |
|  | A75/17 | Wildtype strain | Switzerland | 1999 | AF112189 | 1824 |
|  | 207/00 | Dog | Italy | 2006 | DQ228166 | 1824 |
|  | * | Mink | Denmark | 1997 | Z47759 | 1824 |
|  | liud | Dog | China | 1999 | AF172411 | 1824 |
|  | PDV-2 | Seal | UK | 1995 | X84998 | 1824 |
|  | Onderstepoort | Vaccine | USA | 2001 | AF378705 | 1815 |
|  | Convac vaccine | Vaccine | Sweden | 1991 | Z35493 | 1824 |
|  | CDV3 | Mink | China | 2008 | EU726268 | 1824 |
|  | Recombinant Snyder Hill | Ferret | USA | 2009 | GU138403 | 1824 |
|  | 55L | Dog | Japan | 2009 | AB475099 | 1824 |
|  | 007Lm | Dog | Japan | 2005 | AB212730 | 1824 |
|  | 5VD | Dog | Japan | 2003 | AY297454 | 1824 |
|  | HLJ1 | Dog | China | 2004 | EU743934 | 1824 |
|  | HLJ2 | Fox | China | 2005 | EU743935 | 1824 |
| CDV-F | * | Mink | China | 2008 | KC427278 | 1989 |
|  | HLJ1-06 | Fox | China | 2006 | JX681125 | 1989 |
|  | PS | Dog | China | 2010 | JN896331 | 1989 |
|  | CDV-RD-JL | Raccoon dog | China | 2014 | KJ848781 | 1989 |
|  | TW-HL3 | Dog | China | 2010 | EU192002 | 1989 |
|  | TW-KS15 | Dog | China | 2010 | EU192026 | 1989 |
|  | SC01 | Raccoon dog | China | 2007 | EF596902 | 1989 |
|  | GN | Raccoon dog | China | 2007 | EF596900 | 1989 |
|  | ZD01 | Fox | China | 2007 | EF596904 | 1989 |
|  | 00-2601 | Raccoon | USA | 2004 | AY443350 | 1989 |
|  | 98-2646 | Raccoon | USA | * | AY542312 | 1989 |
|  | 18133 | Dog | USA | * | AY964108 | 1989 |
|  | 25259 | Dog | USA | * | AY964114 | 1989 |
|  | 01-2689 | Raccoon | USA | * | AY649446 | 1989 |
|  | Lion94SNP | African lion | Tanzania | 1994 | JN812977 | 1989 |
|  | R11-CN-114 | Raccoon dog | South Korea | 2011 | JQ327710 | 1989 |
|  | 50Cbl/H | Dog | Japan | * | AB490678 | 2016 |
|  | Vaccine X | Vaccine | Hungary | 2006 | EU072198 | 1989 |
|  | A75/17 | Wildtype strain | Switzerland | 1999 | AF112188 | 1989 |
|  | 171391-513 | Dog | USA | 2004 | KJ123771 | 1989 |
|  | 164071 | Dog | USA | 2004 | EU716337 | 1989 |
|  | Onderstepoort | Vaccine | USA | 2001 | AF378705 | 1989 |
|  | Recombinant Snyder Hill | Ferret | USA | 2009 | GU138403 | 1989 |
|  | Shuskiy | Mink | Kazakhstan | 1989 | HM063009 | 1989 |
|  | CDV3 | Mink | China | 2008 | EU726268 | 1989 |
|  | Giant panda/SX/2014 | Giant panda | China | 2014 | KP793921 | 1989 |
|  | 007Lm/H | Dog | Japan | * | AB490679 | 1989 |
|  | R11-JB-081 | Raccoon dog | South Korea | * | JQ315345 | 1989 |
|  | R11-JB-014 | Raccoon dog | South Korea | * | JQ315341 | 1989 |

Note: * Not available.


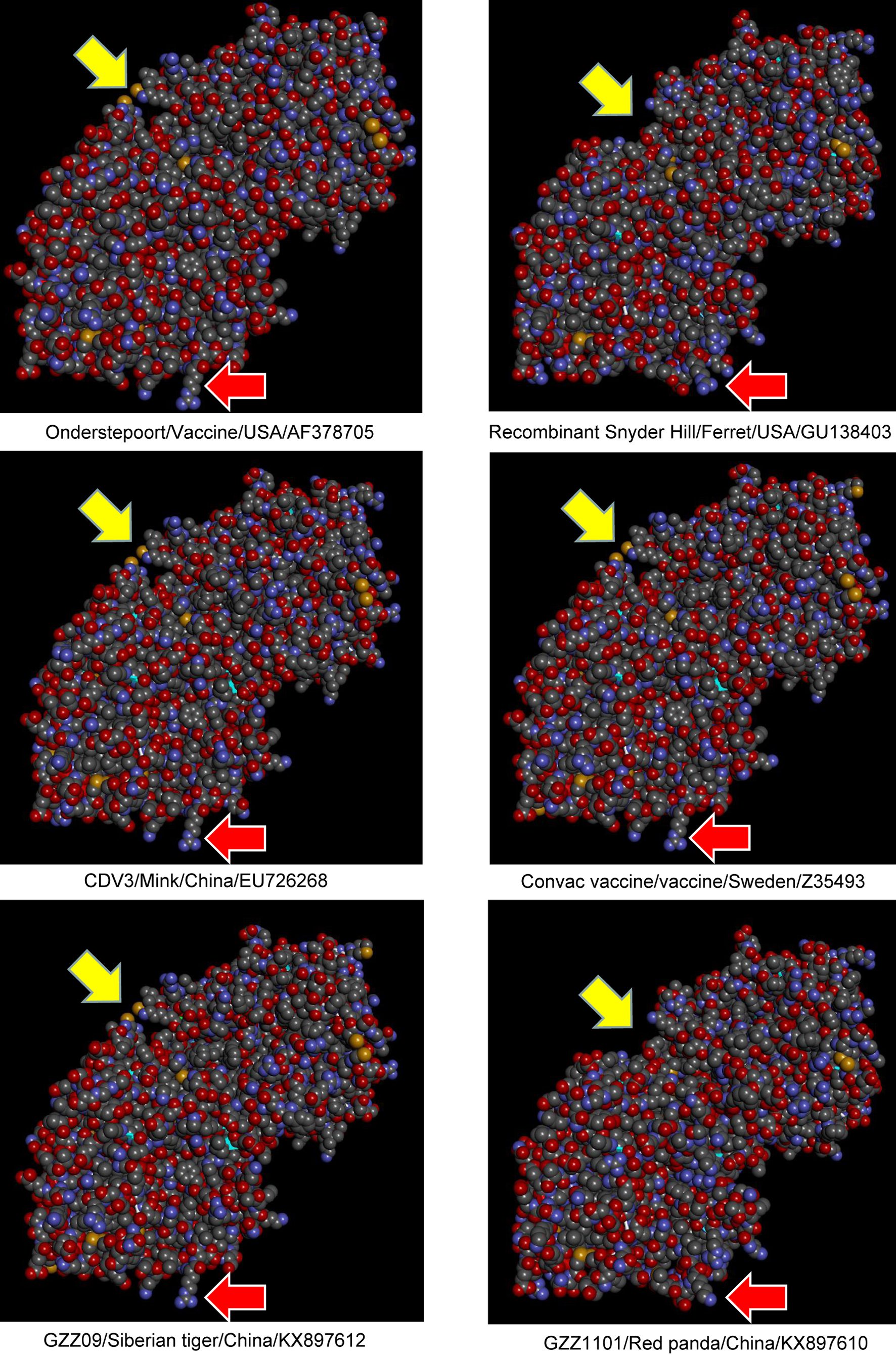


**Supplemental Figure S1** Advanced structural profiles of H protein of Siberian tiger-origin and red panda-origin CDV strains and vaccine strains.


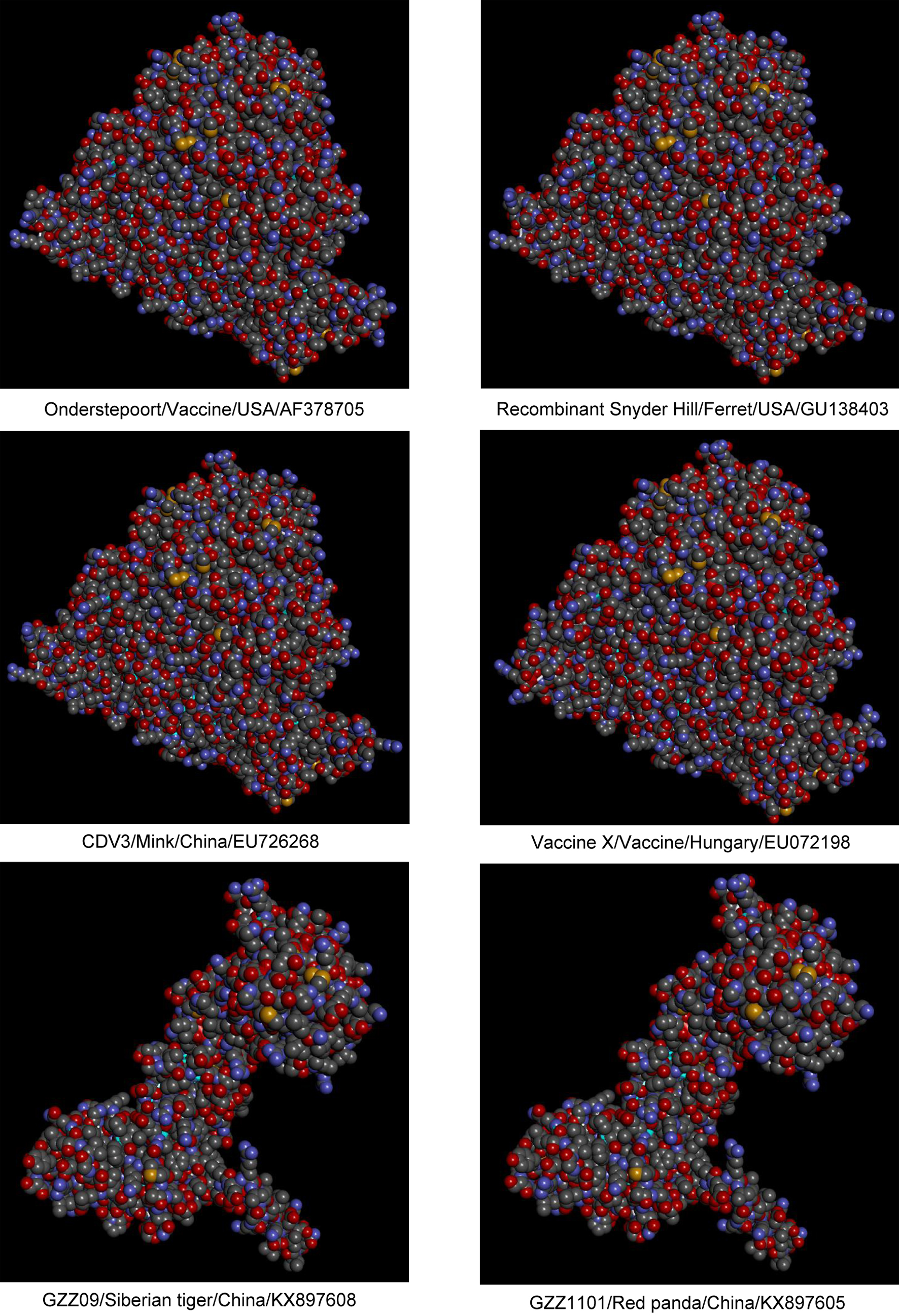


**Supplemental Figure S2** Advanced structural profiles of F protein of Siberian tiger-origin and red panda-origin CDV strains and vaccine strains.
